# Developing standards to support cell technology applications

**DOI:** 10.1111/cpr.13210

**Published:** 2022-03-11

**Authors:** Jiani Cao, Glyn Stacey, Ng Shyh‐Chang, Tongbiao Zhao

**Affiliations:** ^1^ State Key Laboratory of Stem Cell and Reproductive Biology Institute of Zoology, Institute for Stem Cell and Regeneration, Chinese Academy of Sciences Beijing China; ^2^ Beijing Institute for Stem Cell and Regenerative Medicine Beijing China; ^3^ Standards Committee Chinese Society for Stem Cell Research, Chinese Society for Cell Biology Shanghai China; ^4^ The International Stem Cell Banking Initiative Ridge Herts UK

Dear Editor,

Cell lines are important resources in both scientific research and regenerative medicine. Scientific research using cell lines has contributed greatly to the understanding of developmental biology and pathology, providing clues to develop new disease therapies, including cell therapies. However, each cell line has unique characteristics that can be changed by suboptimal cell culture techniques, prolonged passaging or suboptimal culture conditions, some of which can be unpredictable. Contamination, either with microorganisms or with other types of cells, and cell line misidentification can also arise during substandard operations. Clearly, the use of substandard cells would waste time and resources, or worse, generate erroneous data that can be misleading and hamper scientific progress and clinical translation. To address these quality control issues, the development of cell technology standards for both scientific research and clinical applications has great significance. Developing such (inter)guidelines calls for extensive collaborative efforts which may require input from scientists, physicians, regulators, funders, entrepreneurs, and others in the field to develop definitive quality control standards that support scientific research and clinical translation, and bolster the reputation of the entire field.

To fill the current gaps in cell technology standardization, a group called the Standards Committee was founded by the Chinese Society for Stem Cell Research (CSSCR) and the Chinese Society for Cell Biology (CSCB) in 2016, with the aim of developing industry standards that facilitate normalized practices in stem cell research and clinical translation. The Standards Committee published the first stem cell standards in China, T/CSCB 0001 General Requirements for Stem Cell in 2017, and the standards for quality control of human embryonic stem cells, T/CSCB 0002 Human Embryonic Stem Cell in 2019. These two standard guidelines were then fully translated into English and published in 2020 in Cell Proliferation.^1^, ^2^ This year, the Standards Committee has completed and released another six cell technology standards, including T/CSCB 0003 Human Mesenchymal Stem Cell, T/CSCB 0004 Human Hematopoietic Stem/Progenitor Cell, T/CSCB 0005 Human Induced Pluripotent Stem Cell, T/CSCB 0006 Human Retinal Pigment Epithelial Cell, T/CSCB 0007 Human Cardiomyocytes and T/CSCB 0008 Primary Human Hepatocyte. In consultation with the broad community, these technology standards have specified the technical requirements, test methods, test regulations, instructions for use, labelling requirements, packaging requirements, storage requirements and transportation requirements for these six types of cell lines, the English version of which are now published in this *Special Issue: Current developments in the manufacture of pluripotent stem cell‐based medicines: Proceedings of PSConf 2021*.^3^, ^4^, ^5^, ^6^, ^7^, ^8^

These six cell technology standards were directly translated from the Chinese committee standard guidelines. As the early and pilot version of industry standard guidelines, these are intended for early adoption and evaluation across the research industry, and the Standards Committee will seek feedback regularly to ensure their practicability and feasibility. The requirements in these standards should provide a good reference for the development of new industry‐specific standards in the future. Of course, there will be some clauses that may require further refinement and we expect new clauses will be suggested by experts or users in the respective fields to improve and broaden their applications. In line with rapid advancements in cell technologies, the implementation of these standards will involve an iterative process with users and experts, to either introduce new clauses or enhance existing clauses as required, in new editions of these standards in the future. In particular, this process is anticipated to further develop certain specific areas as discussed below:

1. Scope

These standards aim to provide a reliable framework for the production and testing of cell preparations in research and development. These documents are scoped for research use only, and they could also be used as reference guidelines (but not yet standards) for the development of diagnostic and therapeutic human cell preparations, subject to meeting the necessary regulatory requirements in each jurisdiction.

2. Analytical methods

It is important to recognize that versatile methods are applicable for single molecular target measurements, and analytical methods develop quickly in the biotechnology field. The standards mentioned include methods provided in the Annexes that are suggested as examples. Upon verification, alternative methods can be selected and used which provide equivalent or superior data. In addition, new analytical methods can be added according to the actual application status and technical advancement.

3. Passage number

Given that the restricted proliferation capacity of some cell types like hepatocytes, cardiomyocytes, mesenchymal stem cells (MSCs), retinal pigmented epithelial cells and haematopoietic stem/progenitor cells, passage number can be inversely correlated with the function of these types of cells. For example, primary hepatocytes and cardiomyocytes have very limited proliferation capacity, and much of their functionality is usually lost after very limited passaging under the current in vitro culture conditions. MSCs will gradually lose the ability to differentiate and, proliferate as their passage number increase in cell culture,^9^ and it is recommended that MSCs for clinical use should be less than Passages 6.^10^

The surface area/volume of a culture vessel, cell density and how the initial P_0_ is defined are all closely associated with the passage number. Due to variations in local practices, cells with the same nominal passage number from different labs may vary in their actual population doubling number as well as functionality. Due to rapid technological improvements, cell culture methods are being upgraded quickly to prolong the maintenance of the cell function in vitro. Therefore, maximum passage or maximum population doubling numbers are expected to evolve quickly. Thus it is more important to focus on and standardize the cell function evaluation requirements, for example, for the quality control of cells in clinical therapies.

Given these considerations, passage number is recommended to be recorded without mentioning the maximum passage or maximum population doubling number, but instead to incorporate information on functionality quality control.

4. Cell markers and function

The acceptance criteria for requirements on cell markers and functional assays for each cell type is defined according to the minimum requirements of current scientific best practices and international consensus. For example, the MSC definition from the International Mesenchymal Organization and Stem Cell Therapy Committee^11^ is included in our current version of the MSC technology standard. The acceptance criteria for marker gene expression in human iPSCs and ESCs is referenced from the *Consensus Guidance for Banking and Supply of Human Embryonic Stem Cell Lines for Research Purposes* released by the International Stem Cell Banking Initiative (ISCBI), which has been extensively discussed and agreed by experts internationally.^12^, ^13^

The cell technology standards published in this special edition of *Cell Proliferantion* ^3^, ^4^, ^5^, ^6^, ^7^ represent an important first step for improving the reproducibility of scientific and clinical data in the broad field of cell technologies, and bolstering the reputation and credibility of the entire field. Community‐driven standardization and quality control guidelines will support the development of national and international standards for this new industry, and might provide substantial guidance in the establishment of CMC standards for clinical‐grade cell preparations and cell therapies in the future.

## AUTHOR CONTRIBUTIONS

Jiani Cao and Tongbiao Zhao contributed to conception and design. Jiani Cao drafted the manuscript. Glyn Stacey, Ng Shyh‐Chang, Tongbiao Zhao critically read and revised the manuscript.

## Data Availability

Data sharing is not applicable to this article as no new data were created or analyzed in this study.
